# Application of cross-priming amplification (CPA) for detection of fowl adenovirus (FAdV) strains

**DOI:** 10.1007/s00705-015-2355-9

**Published:** 2015-02-06

**Authors:** Jowita Samanta Niczyporuk, Grzegorz Woźniakowski, Elżbieta Samorek-Salamonowicz

**Affiliations:** Department of Poultry Viral Disease, National Veterinary Research Institute, Partyzantów 57 Avenue, 24-100 Pulawy, Poland

## Abstract

Fowl adenoviruses (FAdVs) are widely distributed among chickens. Detection of FAdVs is mainly accomplished by virus isolation, serological assays, various polymerase chain reaction (PCR) assays, and loop-mediated isothermal amplification (LAMP). To increase the diagnostic capacity of currently applied techniques, cross-priming amplification (CPA) for the detection of the FAdV hexon gene was developed. The single CPA assay was optimised to detect all serotypes 1-8a-8b-11 representing the species *Fowl aviadenovirus A-E*. The optimal temperature and incubation time were determined to be 68 °C for 2 h. Using different incubation temperatures, it was possible to differentiate some FAdV serotypes. The results were recorded after addition of SYBR Green I^®^ dye, which produced a greenish fluorescence under UV light. The CPA products separated by gel electrophoresis showed different “ladder-like” patterns for the different serotypes. The assay was specific for all serotypes of FAdV, and no cross-reactivity was observed with members of the genus *Atadenovirus*, duck atadenovirus A (egg drop syndrome virus EDS-76 [EDSV]) or control samples containing Marek’s disease virus (MDV), infectious laryngotracheitis virus (ILTV) or chicken anaemia virus (CAV). The results of the newly developed FAdV-CPA were compared with those of real-time PCR. The sensitivity of CPA was equal to that of real-time PCR and reached 10^−2.0^ TCID_50_, but the CPA method was more rapid and cheaper than the PCR systems. CPA is a highly specific, sensitive, efficient, and rapid tool for detection of all FAdV serotypes. This is the first report on the application of CPA for detection of FAdV strains.

## Introduction

Fowl adenoviruses (FAdVs) are distributed in chicken flocks worldwide [7]. Every year, the number of FAdV infections in birds increases [4–6, 8, 13, 15, 25, 26, 29, 32, 33, 35].

For a long time, the primary role of FAdVs in pathogenesis was not fully clarified, as FAdVs were isolated from both clinically healthy and diseased birds, or together with other immunosuppressive agents (e.g., infectious bursal disease virus [IBDV] and chicken anaemia virus [CAV] [23]). Currently, it is acknowledged that virulent FAdVs belonging to certain serotypes of different species act as a primary pathogens, inducing characteristic clinical signs alone without co-infection with any other pathogens. This knowledge came from the emergence of highly virulent strains during the late 1980s and 1990s (hydropericardium hepatitis syndrome [HHS] outbreaks in Pakistan, spreading to other countries of the world) [7, 10] and successful experimental induction of characteristic signs by inoculation of SPF chickens with strains isolated from field outbreaks. Development of improved methods for the differentiation of FAdV strains has helped to elucidate the pathogenic role of FAdVs causing different clinical syndromes such as inclusion body hepatitis (IBH), HHS, and gizzard erosion and ulceration (GEU) [5, 7, 8, 13].

The clinical picture of IBH is caused predominantly by strains from serotypes FAdV-2 and -11, representing FAdV-D, and serotypes FAdV-8a and -8b, representing FAdV-E. The current perception of the role of pathogenic FAdVs has changed, since some serotypes are able to induce specific clinical signs without co-infection with additional agents. Indeed, some FAdV strains are responsible for the clinical picture of IBH. These etiological agents are predominantly strains belonging to the species *Fowl aviadenovirus D*, representing serotypes FAdV-2, 3, 9, and 11, and the species *Fowl aviadenovirus E*, representing serotypes FAdV-6, 7, 8a, and 8b [2]. Adenoviruses have been isolated from sick birds as well as from birds without any clinical signs of infection [10, 11]. FAdVs may cause disease independently, or they could be one of the factors of multi-aetiological syndromes [2]. Another example is HHS [7], which is caused by the serotype FAdV-4, which belongs to the species *Fowl aviadenovirus C*, and GEU, which is mainly induced by serotype FAdV-1, species *Fowl aviadenovirus A* [8, 30, 31].

FAdVs may also cause immunodeficiency or vaccination failure in chickens [26]. Due to the serious economic loss they are able to cause, FAdVs represent a major concern in the poultry industry.

So far, FAdV detection methods have been based on virus isolation in chicken embryo fibroblasts (CEFs), chicken embryo liver cells (CELs), chicken embryo kidney cells (CEKs), a chicken hepatoma cell line (LMH) [7], or specific-pathogen-free (SPF) chicken embryos [22], or inoculation of SPF chickens with different serotypes. FAdV antibodies can also be detected in serum by serological assays, including agar gel precipitation assay (AGP), virus seroneutralisation (SN), and ELISA [24, 35]. Specific commercial antibodies are available only against FAdV-1, FAdV-3 and FAdV-5, or FAdV of unspecified serotype. Consequently, IgG antibodies against serotype-specific and group-specific antigens of fowl adenovirus serotypes FAdV-2, 3 and 4 have been identified by ELISA [24].

Several PCR assays for the detection of members of different FAdV species and serotypes have been developed in the last 15 years, including PCR combined with restriction enzyme analysis [20]. Methods based on PCR are useful and able to identify several FAdV serotypes [1, 17, 39, 44]. PCR was described for the first time for the detection of pIIIa and pIII genes of serotypes FAdV-8 and FAdV-1 [14], and nested-PCR assays based on the polymerase gene [16, 41] and duplex-PCR assays for detection of serotypes FAdV-1 and FAdV-5 have also been developed [27]. Recently, PCR targeting the hexon gene was developed [18, 20, 21, 37, 44]. The same technique was also used for the detection of haemorrhagic enteritis virus (HEV) and egg drop syndrome virus (EDSV), belonging to the genera *Siadenovirus* and *Atadenovirus*, respectively [11, 18, 19]. In addition, real-time PCR has been shown to be a useful tool for detection of members of the five FAdV species (A-E) [9].

Novel techniques based on isothermal amplification, especially loop-mediated isothermal amplification (LAMP), have been developed previously for the detection of many poultry pathogens, including infectious bronchitis virus (IBV) [2], chicken anaemia virus (CAV) [12], avian influenza virus (AIV) [36], Newcastle disease (NDV) [34], infectious bursal disease (IBDV) [46] and Marek’s disease virus [42, 43]. Recently, the LAMP assay was shown to be a useful and sensitive method for identification of FAdV **[** 26, 28, 40, 45]. Detection of specific products is performed under UV illumination after addition of SYBR Green I^®^ dye, resulting in greenish fluorescence in positive samples. A recent innovation of isothermal amplification methods is cross-priming amplification (CPA), which is primarily invented by Ustar Biotechnologies (Hangzhou, China) and described by Rendong et al. [38]. CPA involves five to six specific primers and leads to cross-priming amplification with an intermediate stage of hairpin-shaped products. Forward cross primer (sense) and reverse cross primer (anti-sense) are aligned with the 5′ end of the sequence. The second defined priming sites of the CPA product are extended by inner and outer primers. After the reaction, the mixture contains several micrograms of amplified DNA products of different length, which are visualised using fluorescent dyes that bind to double-stranded nucleic acids. *Bst* and *Bsm* polymerases have been used previously because of their isothermal polymerase activity. More recently, however, the isothermal polymerase *Gsp*SSD polymerase, isolated from *Geobacillus* sp. has been used due to its improved activity and resistance to reaction inhibitors. So far, the CPA protocol has been adapted mainly for the detection of *Mycobacterium tuberculosis*. A schematic concept of CPA was presented by Rendong et al. [38, 47, 48]. CPA has not been developed so far for the detection of any avian pathogens.

The aim of this study was to develop and optimise CPA using five specific primers corresponding to hypervariable regions HVR-1-4 of the hexon gene, which are the most variable regions of the FAdVs genome.

## Materials and methods

### Standard strains

Standard FAdV (1-8a-8b-11) strains, FAdV-1-CELO, FAdV-2, FAdV-3, FAdV-4, FAdV-5-TIPTON, FAdV-6, FAdV-7, FAdV-8a, FAdV-8b, FAdV-9, FAdV-10, and FAdV-11, representing FAdV species A-E were purchased from Charles River Laboratories (North Franklin, Connecticut, USA) as lyophilised virus stocks. Reference strains were propagated in CEFs according to standard procedure with infectious titres ranging from 10^3.0^ TCID_50_/ml to 10^5.5^ TCID_50_/ml. The viruses were harvested by a triple freezing-thawing procedure and stored at −20 °C.

### Field strains

Thirty adenovirus field strains from our own laboratory collection were used, representing seven serotypes: FAdV-1 (14/08w, 66/09w, 110/10z, 27/10j, 56/11z, and 61/11z), FAdV-2/11j (32/10j), FAdV-4 (31/10z, 62/10z, 64/10j, and 59/11w), FAdV-5-TIPTON (131/10TF, 45/10j, 45/11z, 88/11j, 88/10z, and 55/11z), FAdV-7 (5/10j, 50/03w, 23/07w, 34/08w, 72/08w), FAdV-8a (37/10z, 51/04w, 48/08w, and 6/12j), and FAdV-8b (35/11j, 10/10j, 14/10ja, and 14/10jb) [21]. The strains were isolated from the liver, intestines, gizzard, and bursa of Fabricius of infected chickens displaying anatomo- and histopathological changes characteristic of adenoviral infection. The field isolates were typed and tentatively assigned to serotypes by hexon-loop-1-based sequence analysis

### Other strains

Marek’s disease virus (MDV) Rispens/CVI988 strain, with a titre of 10^3.6^ TCID_50_, was isolated from a commercial vaccine (MSD Animal Health, AA Boxmeer, The Netherlands). Infectious laryngotracheitis virus (ILTV) with a titre of 10^3.0^ TCID_50_, derived from Nobilis ILT vaccine (MSD Animal Health, AA Boxmeer, The Netherlands), chicken anemia virus (CAV) strain 26P4 with a titre of 10^3.0^ TCID_50_, obtained from Nobilis (Intervet Schering**-**Plough, The Netherlands), and egg drop syndrome virus (EDSV) strain A127 with a titre of 10^3.0^ TCID_50_ (Lohmann Animal Health, Germany) were used for the evaluation of CPA specificity.

### Virus propagation

Chicken Embryo Fibroblasts (CEFs) were prepared from 11-day-old SPF chicken embryos (Lohmann Animal Health, Cuxhaven, Germany) according to the standard procedure. The growth medium was Eagle’s medium (MEM) supplemented with 10 % bovine serum and 0.1 % antibiotic mixture (Antibiotic–Antimycotic, Gibco, Paisley, Scotland). The maintenance medium consisted of MEM with 0.1 % antibiotic mixture. A monolayer of CEFs was obtained after about 24 h of incubation at 37.5 °C and 5 % CO_2_ and was inoculated with reference FAdV strains and with homogenates obtained from internal organs of sick chickens. The organ samples were prepared by a triple freezing and thawing procedure, centrifugation, and filtering through filters with 450 nm pore size (Millipore, Billerica, USA). Infected cells were incubated until a cytopathic effect (CPE) was observed in 80 % of the monolayer, and collected supernatants were used for the next passage. The material from the third passage was used for DNA extraction.

### DNA extraction

FAdV DNA was extracted from 200 µl of infected CEF culture using a QIAamp DNA Mini Kit (QIAGEN, Hilden, Germany) according to the manufacturer’s instructions. Negative control DNA for CPA and real-time PCR were extracted from uninfected CEFs. The DNA was stored at −20 °C for the next step of the study as template for CPA.

### PCR

The reaction was carried out in a basic gradient thermocycler (Biometra, Germany) in a final volume of 25 μl of reaction mix. The mixture contained 2.5 μl of PCR buffer, 1 μl of dNTP (10 mM), 1.5 μl of each pair of primers, 4 μl of total DNA isolated from reference strains, and 11.5 μl of sterile water. Pre-denaturation was done at 95 °C for 5 min, followed by 35 cycles of denaturation at 94 °C for 45 s, primers annealing at 61 °C for 1 min, and chain elongation at 72 °C for 2 min, and then a final elongation step was carried out at 72 °C for 10 min.

### Analysis of PCR products

After the amplification reaction, electrophoresis was conducted in a 2 % agarose gel with 1 μg of ethidium bromide per mL in a Mini-Sub Cell (Bio-Rad, USA). Electrophoresis was carried out at 150 V and 80 mA for 50 min in Tris-borate-EDTA buffer, pH 8.2. The sizes of amplified products were compared with a MassRuler 1,031-bp DNA marker (Fermentas). The bands were visualised using a UV transilluminator and then photographed and analysed. The results were considered positive when the products obtained with a specific pair of nucleotide primers had the predicted size.

### Sequencing and phylogenetic analysis

PCR products were purified using a NucleoSpin Extract II Kit (Marcherey-Nagel, France) and then commercially sequenced by GENOMED (Warsaw) using a GS FLX/Titanium sequencer (Roche, Switzerland). Phylogenetic analysis was performed by alignment of the nucleotide sequences of the amplified fragments of the hexon gene originating from the field adenovirus strain FAdV with those of the fowl adenovirus reference sequences from the GenBank database that had been identified according to the general obligatory classifications. A phylogenetic tree was generated by the neighbor-joining method by the p-distance method (on 1000 bootstrapped datasets). Four sequences of reference human adenovirus strains were also used in the dendrogram. The analyses were performed using the computer software MEGA5, Geneious 6, and BLAST. On the basis of this analysis, a phylogenetic tree was constructed and the relationships between the examined adenovirus strains were determined.

### Design of FAdV-CPA primers

**Fig. 1:**
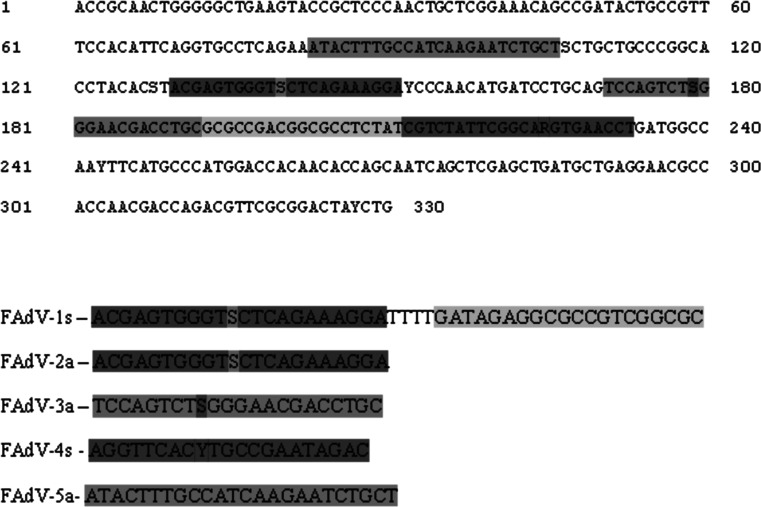
Location of CPA primer sites within the hexon gene of FAdV (GenBank accession number AF339914). 1s, cross-primer; 2a and 3a, inner primers; 4s and 5a, outer primers. The degenerate primer sites are highlighted

The loop-1 region of the hexon gene of FAdV-A reference strain CELO (GenBank accession number AF339914) was compared with other available hexon gene sequences of FAdV reference strains in order to select a conserved sequence about 151 bp long as the detection target. Five primers complementary to the hexon gene sequence of FAdV were designed manually. The locations of the CPA primer sequences in the hexon gene are shown in Fig.1.

### Optimisation of FAdV-CPA

A reaction mix was made on ice in 0.2-ml optical tubes with five complementary primers. Reaction parameters were optimised using Isothermal Mastermix (OptiGene, Horsham, West Sussex, UK) with different primer concentrations: 1s (10–40 pM), 2a and 3a (5–20 pM), 4s, and 5a (2.5–10 pM). The temperature of incubation was optimised at 62 °C, 64 °C, and 68 °C. The last parameter optimised was the incubation time, which ranged from 30 min to 3 h. After incubation, 1 µl of 10,000-fold-diluted SYBR Green^®^ I dye (Invitrogen, Germany) was added to each tube. Positive reactions gave greenish fluorescence under UV light. The CPA products were analysed in agarose gels stained with 2 % GelRed and formed a “ladder-like” pattern with sizes ranging from 80 to 1031 bp when compared to the molecular-length marker GeneRuler™ 100 bp DNA Ladder Plus (Thermo Scientific, Waltham, Massachusetts, USA). Agarose gels were documented and photographed under UV light (GenoSmart Gel documentation system, VWR, Germany). During the optimization, all samples were tested in triplicate.

### Specificity and sensitivity of FAdV-CPA

The specificity of the assay was evaluated using DNA extracted from uninfected CEFs as well as from the MDV Rispens/CVI988, ILTV, CAV, and EDS strains.

The sensitivity of the CPA was determined using tenfold dilutions (10^0^ to 10^−5.0^) of 2 µl DNA extracted from 200 µl of infected cell culture supernatant containing 10^3.0^ TCID_50_/ml of FAdV-A reference strain CELO. The CPA products were then analysed by electrophoresis in 2 % agarose gels. The sensitivity was defined as the highest dilution showing greenish fluorescence. Thirty field strains of FAdV were examined using CPA under conditions optimised with reference strains. The assay was able to detect a 10^−5.0^ dilution of DNA extracted from infected cell culture supernatant containing 10^3.0^ TCID_50_ FAdV-A/CELO.

### Real-time PCR

The primer sequences used for real-time PCR specific for FAdV were Adeno F (5′-AATGTCACNACCGARAAGGC-3′, sense primer) and Adeno R (5′-CBGCBTRCATGTACTGGTA-3′). The primers were designed using Primer 3 software. The real-time PCR primers were based on a loop L1 fragment of the hexon gene and were strictly specific for the individual FAdV serotypes.

Real-time PCR was conducted in an ABI7500 system (Applied Biosystems, Foster City, California, USA) in a final volume of 25 µl containing 12.5 µl of Master Mix SYBR Green 2x (QIAGEN, Hilden, Germany), 1.0 µl of primer Adeno F (40 pM), 1.0 µl of primer Adeno R (40 pM), 8.5 µl of PCR-grade water, and 2.0 µl of DNA template. After a pre-denaturation step at 95 °C for 15 min, 40 cycles with subsequent signal acquisition were conducted at 94 °C for 30 s and 55 °C for 45 s. The programme was completed after a melting curve analysis in the temperature range of 55 °C to 95 °C. The sensitivity of the method was defined based on the amplification curve and cycle threshold value (Ct) detected in tenfold dilutions of DNA extracted from CEFs infected with FAdV-1 reference strain CELO.

## Results

**Fig. 2:**
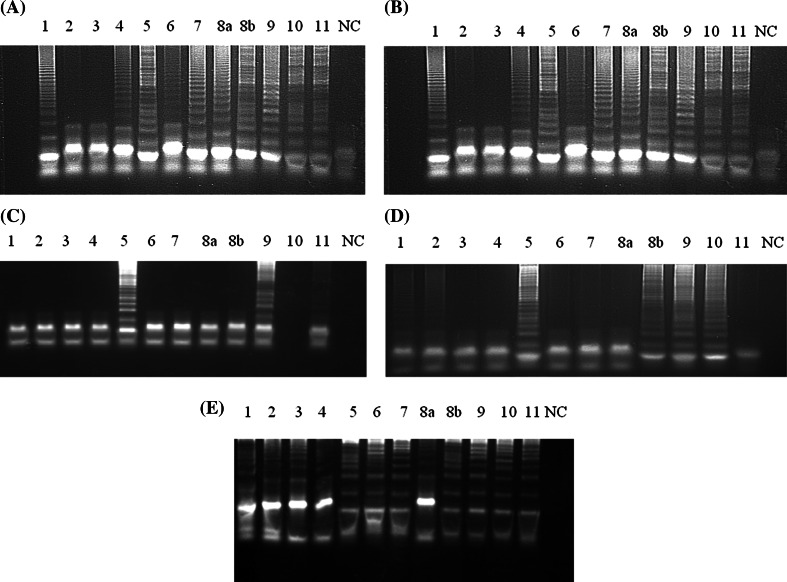
CPA temperature and primer optimisation. A and B, 62 °C; C, 64 °C; D and E, 68 °C. The primer concentrations were as follows: (A) 1s, 40 pM, 2s, 40 pM, 3s, 20 pM, 4s, 20 pM, 5a, 10 pM. (B) 1s, 80 pM, 2s, 40 pM, 3s, 40 pM, 4s, 20 pM, 5a, 20 pM. (C) 1s, 80 pM, 2s, 40 pM, 3s, 40 pM, 4s, 20 pM, 5a, 20 pM. (D) 1s, 80 pM, 2s, 40 pM, 3s, 40 pM, 4s, 20 pM, 5a, 20 pM. (E) 1s, 40 pM, 2s, 20 pM, 3s, 20 pM, 4s, 10 pM, 5a, 10 pM. The numbers indicate the serotypes of FAdV. NC, DNA extracted from uninfected SPF chicken embryo fibroblasts (CEFs)

**Fig. 3:**
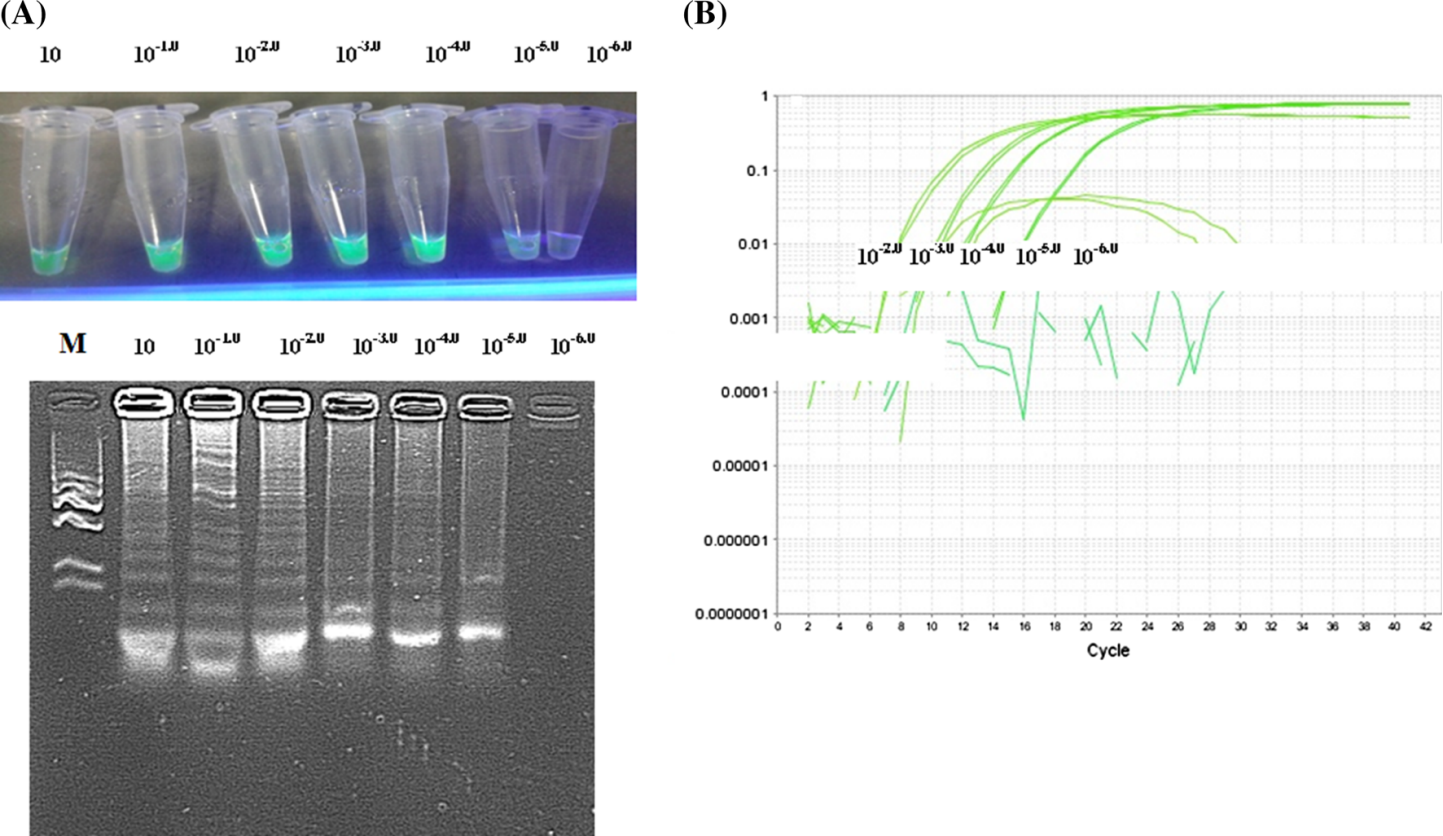
Sensitivity of CPA vs. real-time PCR. (A) The upper panel shows CPA results observed under UV light, while the lower panel shows results obtained after gel electrophoresis. (B) real-time PCR specific for the hexon gene of FAdV. Amplification was done using samples containing dilutions of the FAdV-1 standard strain. The concentration of the virus in each sample is given in TCID_50_/ml (tissue culture infectious dose per ml). M, molecular length marker GeneRuler™ 100 bp DNA Ladder Plus (Thermo Scientific, Waltham, Massachusetts, USA). ΔRn is the change in the fluorescent signal during the real-time PCR cycles

CPA optimisation was done using standard FAdV (1-8a-8b-11) strains. At first, different primer concentrations (5 pM, 10 pM, 20 pM, and 40 pM) were tested at 62 °C, 64 °C, and 68 °C. The optimal reaction time was 2 h (data not shown). The results are presented in Fig. 2A–E. At 62 °C, the primer concentrations were as follows: 1s, 40 pM; 2a, 40 pM; 3a, 20 pM; 4s, 20 pM; 5a, 10 pM. Positive results were obtained for all serotypes, except strains representing serotypes FAdV-2 and FAdV-3 (Fig. 2A). Similar results were obtained at the same temperature with the following primer concentrations: 1s, 80 pM; 2a, 40 pM; 3a, 40 pM; 4s, 20 pM; and 5a, 20 pM (Fig. 2B). At 64 °C, the primer concentrations were as follows: 1s, 80 pM; 2a, 40 pM; 3a, 40 pM; 4s, 20 pM; 5a, 20 pM. Positive results were obtained only with samples containing strains representing the FAdV-5 and FAdV-9 serotypes (Fig. 2C). Next, the temperature of the reaction was increased to 68 °C and the primer concentrations were as follows: 1s, 20 pM; 2a and 3a, 10 pM; 4s and 5a, 10 pM. Positive results were obtained in samples containing serotypes FAdV-5, FAdV-8b, FAdV-9, and FAdV-10 (Fig. 2D). Finally, the optimal conditions of the reaction were incubation for 2 h at 68 °C with the following primer concentrations: 1s, 40 pM; 2a and 3a, 20 pM; 4s and 5a, 10 pM. The optimised conditions allowed the detection of reference strains representing all 12 FAdV serotypes (Fig. 2E). The amplicons displayed different “ladder-like” patterns of bands after gel electrophoresis. No positive signal was observed in the case of DNA samples extracted from uninfected CEFs or other viruses used as negative controls. The detection limit of CPA was 10^−5.0^ TCID_50_/ml for the FAdV-1 serotype with a titer of 10^3.0^ TCID_50_ (Fig. 3A). The sensitivity was similar in the case of the real-time PCR (Fig. 3B).

**Fig. 4:**
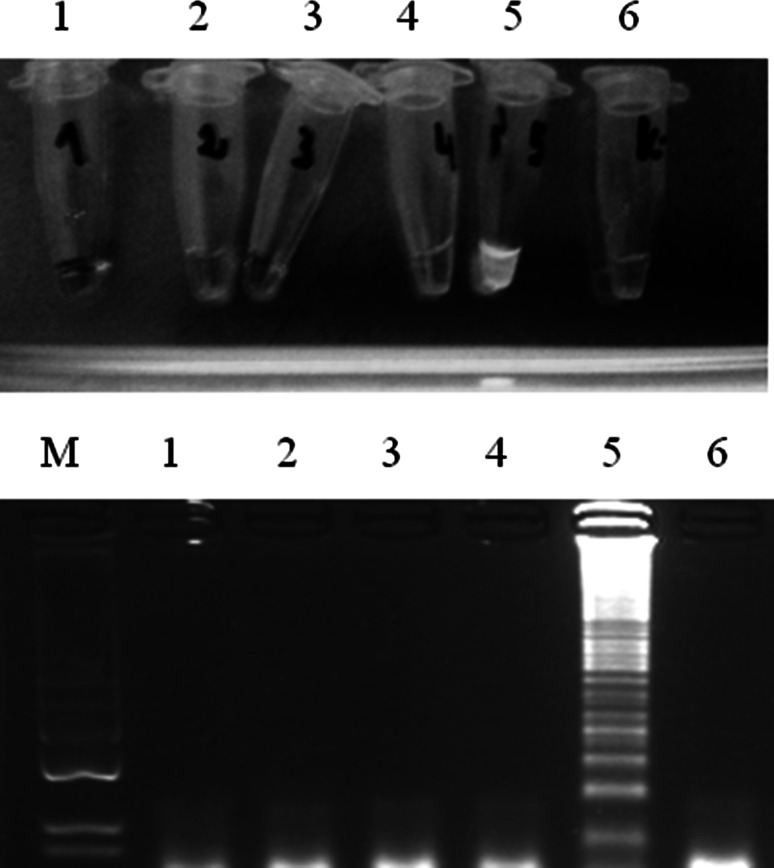
Specificity of CPA for detection of FAdVs. Positive samples yielded greenish fluorescence under UV light and a “ladder-like” pattern of amplification products after agarose gel electrophoresis. 1, DNA extracted from uninfected SPF chicken embryo fibroblasts (CEF); 2, Rispens/CVI988; 3, EDS; 4, ILTV; 5, FAdV-1; 6, CAV. M, molecular length marker GeneRuler™ 100 bp DNA Ladder Plus (Thermo Scientific, Waltham, Massachusetts, USA)

The specificity of FAdV-CPA was demonstrated using DNA extracted from Rispens/CVI988, EDS, ILTV, and CAV strains. All samples containing DNA of these viruses were negative (Fig. 4).

**Fig. 5:**
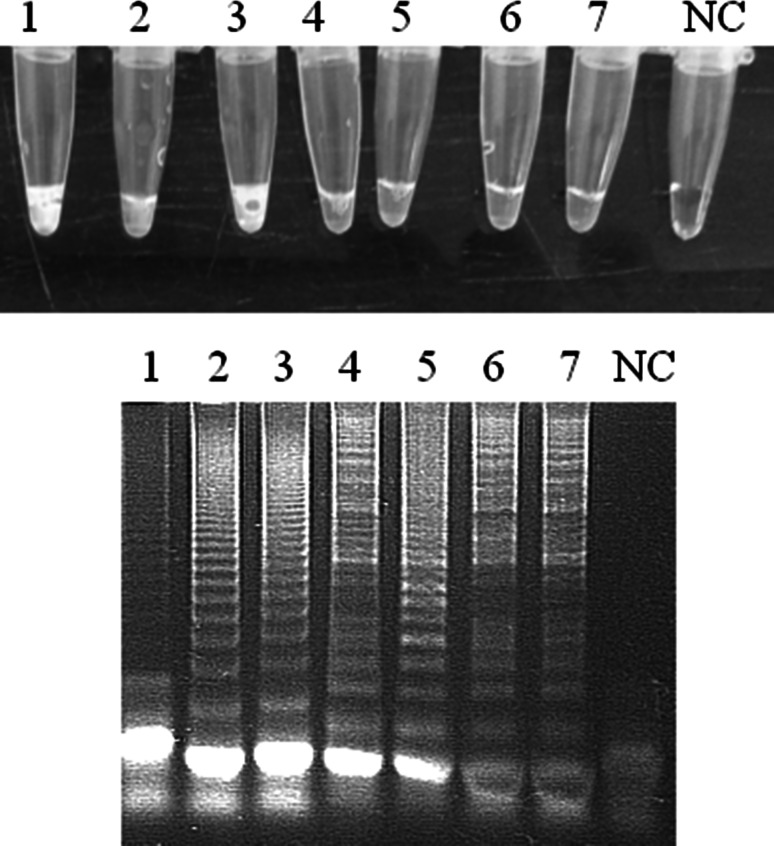
An example of detection of FAdV field strains using CPA. Positive samples yielded greenish fluorescence under UV illumination, and gel electrophoresis of CPA products resulted in a “ladder-like” pattern of bands. 1, 14/08w; 2, 32/10j; 3, 31/10z; 4, 131/10TF; 5, 5/10j; 6, 37/10z; 7, 35/11j; NC, DNA extracted from uninfected SPF chicken embryo fibroblasts (CEFs)

During the next step of this study, we applied the optimised CPA assay to detect 30 field adenovirus strains from the collection of the Department of Poultry Viral Diseases (National Veterinary Research Institute, Pulawy, Poland), representing to seven different serotypes (FAdV-1, FAdV-2/11, FAdV-4, FAdV-5, FAdV-7, FAdV-8a, and FAdV-8b). The DNA of all investigated FAdV field strains was successfully detected by the CPA. A greenish fluorescence was observed in positive samples under UV light, as was the characteristic “ladder-like” pattern of amplification products after gel electrophoresis (Fig. 5).

## Discussion

Early diagnosis is crucial for controlling FAdV infections. Classical virological methods such as virus isolation in SPF chicken embryos or in cell culture are laborious and time-consuming. In spite of this, they are still used as a “gold standard” [22].

In recent years, molecular biological methods based on PCR have been frequently used for detection and identification of adenoviruses [1, 17, 19–22, 26–28]. Our study was conducted to develop and optimise a specific, sensitive, and rapid tool for the detection and identification of FAdV strains. A variable region of the FAdV hexon loop-1 was selected as the target region for amplification, using CPA primers corresponding to a region with a high degree of conservation between all FAdV serotypes.

The CPA method described here relies on the isothermal amplification of nucleic acids. CPA is catalysed by *Bsm*, *Bst* or GspSSD polymerase and does not require an initial denaturation step or other enzymes [38]. The results of this study show that CPA is a specific detection method for all FAdV serotypes and could potentially be used to differentiate between different serotypes of the virus based on the observation that each serotype yields a characteristic ladder-like pattern of bands. CPA can be performed without any special laboratory equipment. The optimised CPA method was used to detect 30 field adenovirus strains representing seven serotypes that are found on poultry farms in Poland. Using the CPA method under different reaction conditions it was possible to differentiate particular serotypes of FAdV. The CPA, which could detect 10^−5.0^ TCID_50_/ml of virus with a titer of 10^3.0^ TCID_50_, is more sensitive than a previously described FAdV-LAMP, which could detect 10^−4.0^ TCID_50_/ml of virus with a titer of 10^3.0^ TCID_50_ [26]. Moreover, the CPA has the same sensitivity as a previously reported real-time PCR [26]. This is in agreement with an earlier study showing CPA and real-time PCR to have similar sensitivity for detection of thrombocytopenia syndrome virus [3]. The CPA reaction time was 2 h and allowed up to 100 copies of the virus to be detected. It therefore seems to be highly suitable for detection of FAdV DNA under field conditions and thus has great diagnostic value.

In conclusion, for the first time, we have developed and evaluated a CPA for rapid identification of FAdVs, a viral pathogen that is responsible for an economically important disease of chickens. The CPA was found to be a rapid and very sensitive tool that can be used by poorly equipped laboratories. The evaluated CPA method has the potential to be used even by mobile laboratories and small veterinary diagnostic units.

## References

[1] Caterina KM, Frasca S, Girshick T, Khan MI (2004). Development of a multiplex PCR for detection of avian adenovirus, avian reovirus, infectious bursal disease virus, and chicken anemia virus. Mol Cell Probes.

[2] Chen HT, Zhang J, Ma YP, Ma LN, Ding YZ, Liu XT, Cai XP, Ma LQ, Zhang YG, Liu YS (2010). Reverse transcription loop-mediated isothermal amplification for the rapid detection of infectious bronchitis virus in infected chicken tissues. Mol Cell Probe.

[3] Cui L, Ge Y, Qi X, Xu G, Li H, Zhao K, Wu B, Shi Z, Guo X, Hu L, You Q, Zhang LH, Freiberg AN, Yu X, Wang H, Zhou M, Tang YW (2012). Detection of severe fever with thrombocytopenia syndrome virus by reverse transcription–cross-priming amplification coupled with vertical flow visualization. J Clin Microbiol.

[4] Dar A, Gomis S, Shirley I, Mutwiri G, Brownlie R, Potter A, Gerdts V, Tikoo SK (2012). Pathotypic and molecular characterization of fowl adenovirus associated with inclusion body hepatitis in Saskatchewan chickens. Avian Dis.

[5] Erny KM, Barr DA, Fahey KJ (1991). Molecular characterization of highly virulent fowl adenoviruses associated with outbreaks of inclusion body hepatitis. Avian Pathol.

[6] Eregae ME, Dewey CE, McEwen SA, Ouckama R, Ojkić D, Guerin MT (2001). 4) Flock prevalence of exposure to avian adeno-associated virus, chicken anemia virus, fowl adenovirus, and infectious bursal disease virus among Ontario broiler chicken flocks. Avian Dis.

[7] Fitzgerald SD, Saif YM, Fadly AM, Glisson JR, McDougald LR, Nolan LK, Swayne DE (2008). Adenovirus infections. Diseases of Poultry.

[8] Gjevre AG, Kaldusdal M, Eriksen GS (2013). Gizzard erosion and ulceration syndrome in chickens and turkeys: a review of causal or predisposing factors. Avian Pathol.

[9] Günes A, Marek A, Grafl B, Berger E, Hess M (2012). Real-time PCR assay for universal detection and quantitation of all five species of fowl adenoviruses (FAdV-A to FAdV-E). J Virol Methods.

[10] Harrach B, Kaján GL, Tidona CA, Darai G (2011). Aviadenovirus. *Adenoviridae*. Springer Index of Viruses.

[11] Hess M, Raue R, Hafez HM (1999). PCR for specific detection of haemorrhagic enteritis virus of turkeys, an avian adenovirus. J Virol Methods.

[12] Huang CH, Lai GH, Lee MS, Lin WH, Lien YY, Hsueh SC, Kao JY, Chang WT, Lu TC, Lin WN, Chen HJ, Lee MS (2010). Development and evaluation of a loop-mediated isothermal amplification assay for rapid detection of chicken anaemia virus. J Appl Microbiol.

[13] Jensen EL, Villegas P (2005). Inclusion body hepatitis: control in breeder and broiler chickens. Avia Tech Eric.

[14] Jiang P, Ojkic D, Tuboly T, Huber P, Nagy E (1999). Application of the polymerase chain reaction to detect fowl adenoviruses. Can J Vet Res.

[15] Kajan GL, Kecskemeti S, Harrach B, Benko M (2013). Molecular typing of fowl adenoviruses, isolated in Hungary recently, reveals high diversity. Vet Microbiol.

[16] Kaján GL, Sameti S, Benko M (2011). Partial sequence of the DNA-dependent DNA polymerase gene of fowl adenoviruses: a reference panel for a general diagnostic PCR in poultry. Acta Vet Hung.

[17] Marek A, Günes A, Schulz E, Hess M (2010). Classification of fowl adenoviruses by use of phylogenetic analysis and high-resolution melting-curve analysis of the hexon L1 gene region. J Virol Methods.

[18] Mase M, Mitake H, Inoue T, Imada T (2009). Identification of group I–III avian adenovirus by PCR coupled with direct sequencing of the hexon gene. J Vet Med Sci.

[19] Mazur-Lech B, Koncicki A, Janta M (2009). Wykorzystanie łańcuchowej reakcji polimerazy (PCR) do wykrywania i określania rozprzestrzeniania się wirusa krwotocznego zapalenia jelit w organizmie indyków. Med Weter.

[20] Meulemans G, Boschmans M, Van Den Berg TP, Decaesstecker M (2001). Polymerase chain reaction combined with restriction enzyme analysis for detection and differentiation of fowl adenoviruses. Avian Pathol.

[21] Meulemans G, Couvreur B, Decaesstecker M, Boschmans M, Van den Berg TP (2004). Phylogenetic analysis of fowl adenoviruses. Avian Pathol.

[22] McConnell BA, Fitzgerald AS, Saif YM (2008). Group I adenovirus infections. Diseases of poultry.

[23] McFerran JB, Adair BM (1977). Avian adenoviruses: a review. Avian Pathol.

[24] Mockett AP, Cook JK (1983). The use of an enzyme-linked immunosorbent assay to detect IgG antibodies to serotype-specific and group-specific antigens of fowl adenovirus serotypes 2, 3 and 4. J Virol Methods.

[25] Nakamura K, Mase M, Yamamoto Y, Takizawa K, Kabeya M, Wakuda T, Matsuda M, Chikuba T, Yamamoto Y, Ohyama T, Takahashi K, Sato N, Akiyama N, Honma H, Imai K (2011). Inclusion body hepatitis caused by fowl adenovirus in broiler chickens in Japan, 2009–2010. Avian Dis.

[26] Niczyporuk JS (2014) Molecular characteristic on occurrence of fowl adenovirus field strains and effect of efficiency on prophylactic vaccinations against Marek’s disease. Doctoral dissertation, National Veterinary Research Institute, Pulawy

[27] Niczyporuk JS, Samorek-Salamonowicz E, Czekaj H (2010). Incidence and detection of aviadenoviruses of serotypes 1 and 5 in poultry by PCR and duplex PCR. Bull Vet Inst Pulawy.

[28] Notomi T, Okayama H, Masubuchi H, Yonekawa T, Watanabe K, Amino N, Hase T (2000). Loop-mediated isothermal amplification of DNA. Nucleic Acids Res.

[29] Ojkic D, Krell PJ, Tuboly T, Nagy E (2008). Characterization of fowl adenoviruses isolated in Ontario and Quebec, Canada. Can J Vet Res.

[30] Okuda Y, Ono M, Shibata I, Sato S, Akashi H (2006). Comparison of the polymerase chain reaction restriction fragment length polymorphism pattern of the fiber gene and pathogenicity of serotype-1 fowl adenovirus isolates from gizzard erosions and from feces of clinically healthy chickens in Japan. J Vet Diagn Invest.

[31] Okuda Y, Ono M, Shibata I, Sato S (2004). Pathogenicity of serotype 8 fowl adenovirus isolated from gizzard erosions of slaughtered broiler chickens. J Vet Med Sci.

[32] Ono M, Okuda Y, Yazawa S, Shibata I, Tanimura N, Kimura K, Haritani M, Mase M, Sato S (2001). Epizootic outbreaks of gizzard erosion associated with adenovirus infection in chickens. Avian Dis.

[33] Pierson F, Fitzgerald S (2008) Hemorrhagic enteritis and related infections. In: Saif YM, Fadly AM, Glisson JR, McDougland LR, Nolan LK, Swayne DE (eds) Diseases of poultry, 12th edn. Blackwell Publishing Professional Ames, Iowa, pp 276–287

[34] Pham HM, Nakajima C, Ohashi K, Onuma M (2005). Loop-mediated isothermal amplification for rapid detection of Newcastle disease virus. J Clin Microbiol.

[35] Philippe C, Grgic H, Ojkic D, Nagy E (2007). Serologic monitoring of a broiler breeder flock previously affected by inclusion body hepatitis and testing of the progeny for vertical transmission of fowl adenoviruses. Can J Vet Res.

[36] Postel A, Letzel T, Frischmann S, Grund C, Beer M, Harder T (2010). Evaluation of two commercial loop-mediated isothermal amplification assays for detection of avian influenza H5 and H7 hemagglutinin genes. J Vet Diagn Invest.

[37] Raue R, Hess M (1998). Hexon based PCRs combined with restriction enzyme analysis for rapid detection and differentiation of fowl adenoviruses and egg drop syndrome virus. J Virol Methods.

[38] Rendong F, Xia L, Lin H, Qimin Y, Jing L, Jie W, Peng X, Huayan Z, Ying L, Jian M, Qian G (2009). Cross-priming amplification for rapid detection of *Mycobacterium tuberculosis* in sputum specimens. J Clin Microbiol.

[39] Singh A, Oberoi MS, Grewal GS, Hafez HM, Hess M (2002). The use of PCR combined with restriction enzyme analysis to characterize fowl adenovirus field isolates from northern India. Vet Res Commun.

[40] Tomita N, Mori Y, Kanda H, Notomi T (2008). Loop-mediated isothermal amplification (LAMP) of gene sequences and simple visual detection of products. Nat Protoc.

[41] Wellehan JF, Johnson AJ, Harrach B, Benko M, Pessier AP, Johnson CM, Garner MM, Childress A, Jacobson ER (2004). Detection and analysis of six lizard adenoviruses by consensus primer PCR provides further evidence of a reptilian origin for the atadenoviruses. J Virol.

[42] Woźniakowski GJ, Samorek-Salamonowicz E, Kozdruń W (2011). Rapid detection of Marek’s disease virus in feather follicles by loop-mediated amplification (LAMP). Avian Dis.

[43] Woźniakowski G, Samorek-Salamonowicz E (2014). First survey of the occurrence of duck enteritis virus (DEV) in free-ranging Polish water birds. Arch Virol.

[44] Xie Z, Fadl AA, Girshick T, Khan MI (1999). Detection of avian adenovirus by polymerase chain reaction. Avian Dis.

[45] Xie Z, Tang Y, Fan Q, Liu J, Pang Y, Deng X, Xie Z, Peng Y, Xie L, Khan MI (2011). Rapid detection of group I avian adenoviruses by a loop-mediated isothermal amplification. Avian Dis.

[46] Xu J, Zhang Z, Yin Y, Cui S, Xu S, Guo Y, Li J, Wang J, Liu X, Han L (2009). Development of reverse-transcription loop-mediated isothermal amplification for the detection of infectious bursal disease virus. J Virol Meth.

[47] Yang HL, Huang J, Yang B, Liu F, Zhang QL (2014). The establishment and application of isothermal cross-priming amplification techniques in detecting penned shrimp white spot syndrome virus. Lett Appl Microbiol.

[48] Zhang F, Wang L, Fan K, Wu J, Ying Y (2014). The detection of T-Nos, a genetic element present in GMOs, by cross-priming isothermal amplification with real-time fluorescence. Anal Bioanal Chem.

